# Supplementary figures and images for: Pathway‐extended gene expression signatures integrate novel biomarkers that improve predictions of patient responses to kinase inhibitors
Source: MedComm (2020). 2020 Dec 10;1(3):311–27. doi: 10.1002/mco2.46 (PMC8491218; doi:10.1002/mco2.46)

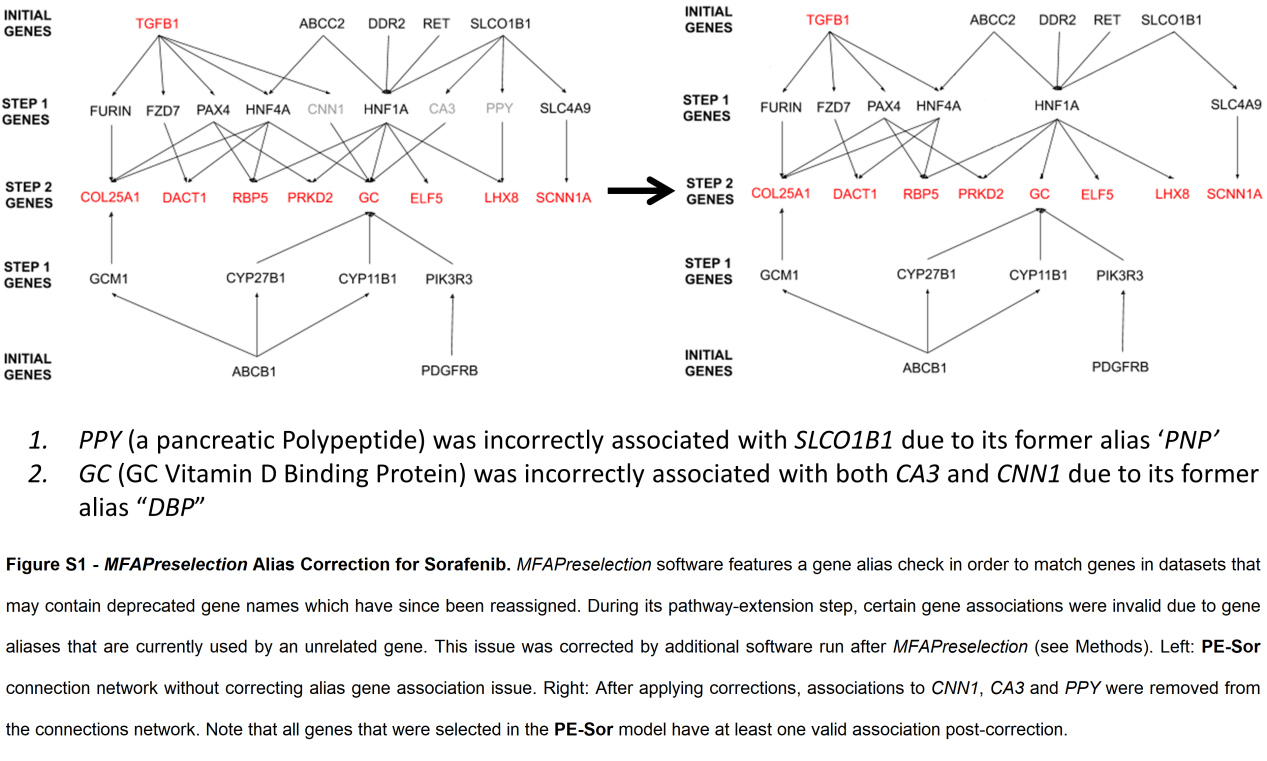


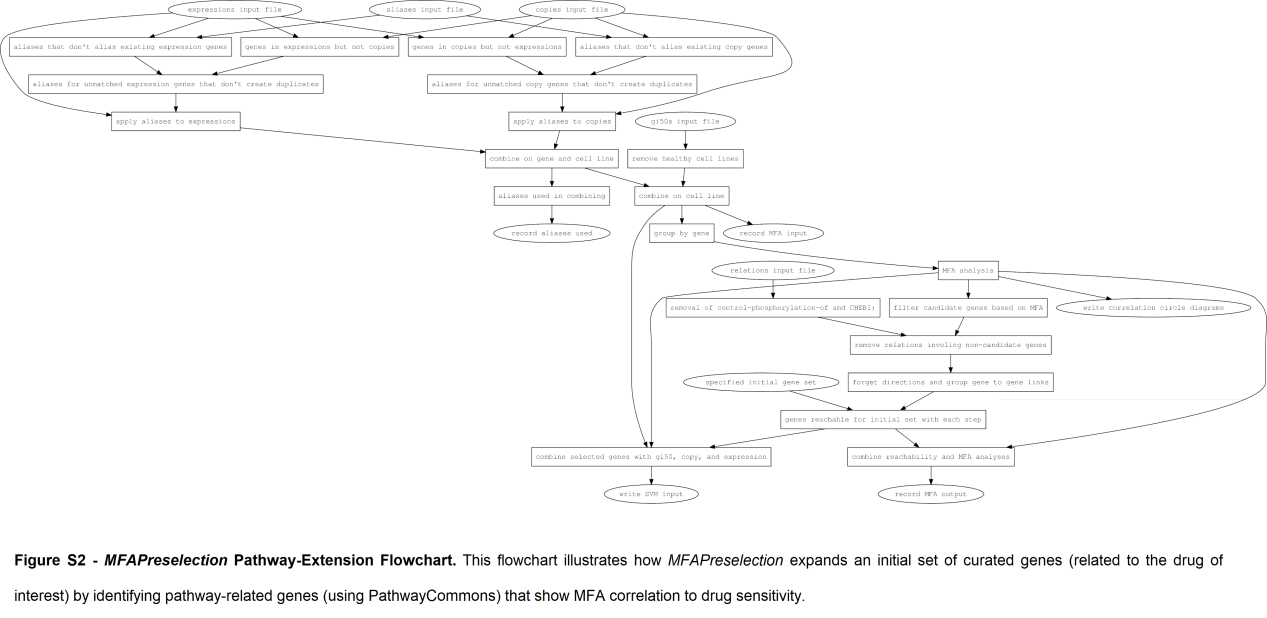

Supplement: Supplementary file 1 — Figure S1‐S2 [file MCO2-1-311-s002.docx]
